# Functional consequences of the oligomeric assembly of Proteorhodopsin

**DOI:** 10.1016/j.jmb.2015.01.004

**Published:** 2015-01-15

**Authors:** Sunyia Hussain, Maia Kinnebrew, Nicole S. Schonenbach, Emily Aye, Songi Han

**Affiliations:** a Department of Chemical Engineering, University of California, Santa Barbara, CA, 93106-5080; b College of Creative Studies, Biology Department, University of California, Santa Barbara, CA, 93106-6110; c Department of Chemistry, University of California, Santa Barbara, CA, 93106-9510

**Keywords:** oligomers, transmembrane proteins, electron paramagnetic resonance, protein-detergent complex

## Abstract

The plasma membrane is the crucial interface between the cell and its exterior, packed with embedded proteins experiencing simultaneous protein-protein and protein-membrane interactions. A prominent example of cell membrane complexity is the assembly of transmembrane proteins into oligomeric structures, with potential functional consequences that are not well understood. From the study of proteorhodopsin (PR), a prototypical seven-transmembrane light-driven bacterial proton pump, we find evidence that the inter-protein interaction modulated by self-association yields functional changes observable from the protein interior. We also demonstrate that the oligomer is likely a physiologically relevant form of PR, as crosslinking of recombinantly expressed PR reveals an oligomeric population within the *E. coli* membrane (putatively hexameric). Upon chromatographic isolation of oligomeric and monomeric PR in surfactant micelles, the oligomer exhibits distinctly different optical absorption properties from monomeric PR, as reflected in a prominent decrease in the pKa of the primary proton acceptor residue (D97) and slowing of the light-driven conformational change. These functional effects are predominantly determined by specific PR-PR contacts over nonspecific surfactant interactions. Interestingly, varying the surfactant type alters the population of oligomeric states as well as the proximity of proteins within an oligomer, as determined by sparse electron paramagnetic resonance (EPR) distance measurements. Nevertheless, the dynamic surfactant environment retains the key function-tuning property exerted by oligomeric contacts. A potentially general design principle for transmembrane protein function tuning emerges from this work, one that hinges on specific oligomeric contacts that can be modulated by protein expression or membrane composition.

## INTRODUCTION

The cell membrane is a hydrophobic barrier that is vital to life, studded with proteins that fulfill the important role of relaying substances and information from the cell’s environment to its interior and vice versa. Since transmembrane proteins occupy a significant portion of the cell surface (from 20–80%^1^), there is a need to understand whether there is a direct functional effect of their assembly into dimers or higher-order oligomers. The association of multiple transmembrane helices within the membrane has been hypothesized to be a general mode of membrane protein assembly^2^ and the analogous formation of oligomeric structures has been experimentally found in archaea, bacteria, and eukaryotes.^3; 4; 5^ Some valuable molecular-level guidelines for the formation of transmembrane protein-protein contacts have emerged from prior structural studies.^6; 7; 8^

Nevertheless, insight into the specific *functional impact* of oligomerization remains elusive, particularly for large membrane proteins with multiple membrane-spanning regions. Aside from the difficulties inherent in the isolation or crystallization of specific oligomeric forms, there are many examples where the monomer is functional by itself, confounding any reason for oligomerization beyond simple structural stability.^9^ Also, the complexity of the membrane environment results in an array of variables that could influence membrane protein structure and function, including both protein-lipid and protein-protein interactions. Thus, any dominant and direct functional effect of transmembrane protein oligomerization can be difficult to separate from the other coexisting, and potentially correlated, environmental factors, especially if the oligomeric contacts are weak.

We seek to evaluate the functional consequences of oligomer formation by (1) choosing a transmembrane protein system that makes oligomeric contacts and is amenable to function studies and (2) implementing an experimental approach to systematically delineate the functional influences of protein-protein interactions due to the underlying oligomeric structure. Here, we investigate the function *tuning* role of oligomerization by examining the self-association of a prototypical seven-helical transmembrane (7TM) protein, the photoactive green-light-absorbing Proteorhodopsin (PR). The PR monomer by itself has all the components (i.e. proton transporting residues) necessary to fulfill its verified function as a proton pump. Yet, previous studies have found PR to exist in homo-oligomeric forms by an array of methods and membrane-mimetic systems,^10,11,12; 13^ most recently x-ray crystallography of blue-absorbing PR^14^ and pulsed EPR analysis of distances across the oligomeric interface.^15^ All of these studies indicate a propensity of PR to oligomerize and contribute structural information, but do not directly elucidate a possible functional influence, let alone the physiological relevance and rationale for the existence of an oligomeric structure. Although the PR gene is found abundantly in marine proteobacteria, these organisms have not been widely cultured aside from a few cases,^16; 17; 18^ such that the native oligomeric form or distribution of PR is unknown.

Different oligomeric species of membrane proteins are often not separated from one another, which is necessary to deconvolute the specific influence of the oligomeric protein-protein interaction over other variables, such as lipid or surfactant effects. Crucially, it has been shown that a oligomeric assembly of PR can be captured within micelles of the nonionic n-dodecyl-β-D-maltoside (DDM) surfactant by separation from lower-order populations using size-exclusion chromatography (SEC), a fast protein liquid chromatography (FPLC) method.^11^ Our study utilizes SEC and introduces alternative surfactant formulations to isolate the monomer and oligomer populations of PR. The distinct characteristics of these isolated oligomeric states can then be extracted by exploiting the light-absorbing property of PR, which permits optical absorption spectroscopy to read out functional attributes. PR is known to actively transport protons upon photoactivation,^19^ creating a pH gradient across the native cell membrane to store solar energy in a biologically utilizable form. This is mediated by the light-induced isomerization of a covalently bound retinal molecule that triggers a series of spectroscopically observable conformational changes (photocycle intermediates) to drive the active transport of a proton from the interior of the cell to the exterior.

The experiments presented here demonstrate that the oligomeric form of PR is a physiologically relevant structure that occurs in the bacterial lipid membrane and has a direct effect on function. Firstly, chemical crosslinking of PR expressed in *E. coli* membranes establishes that an oligomeric form of PR is present in the bilayer. Furthermore, key functional properties of PR, such as pH-dependent light absorption and conformational dynamics of a key photoactivation intermediate, are found to dramatically vary between the monomeric and oligomeric forms of PR within a micelle environment. The influence of specific surfactants was tested using both the nonionic sugar-based DDM and the zwitterionic single-chain dodecylphosphocholine (DPC) surfactant, and found to be minor relative to the effect of oligomerization. This is particularly remarkable, as the nature of the membrane-mimetic micelle was found to change the populations of oligomeric complexes as well as the structural quality of protomer packing within the oligomer, as evaluated by SEC and site-specific electron paramagnetic resonance (EPR) line shape analysis, respectively. Altogether, our study establishes specific protein-protein interactions within the membrane to be direct modulators or even determinants of transmembrane protein function.

## RESULTS

### Evidence for oligomeric PR in the *E. coli* membrane by chemical crosslinking

**Figure 1:**
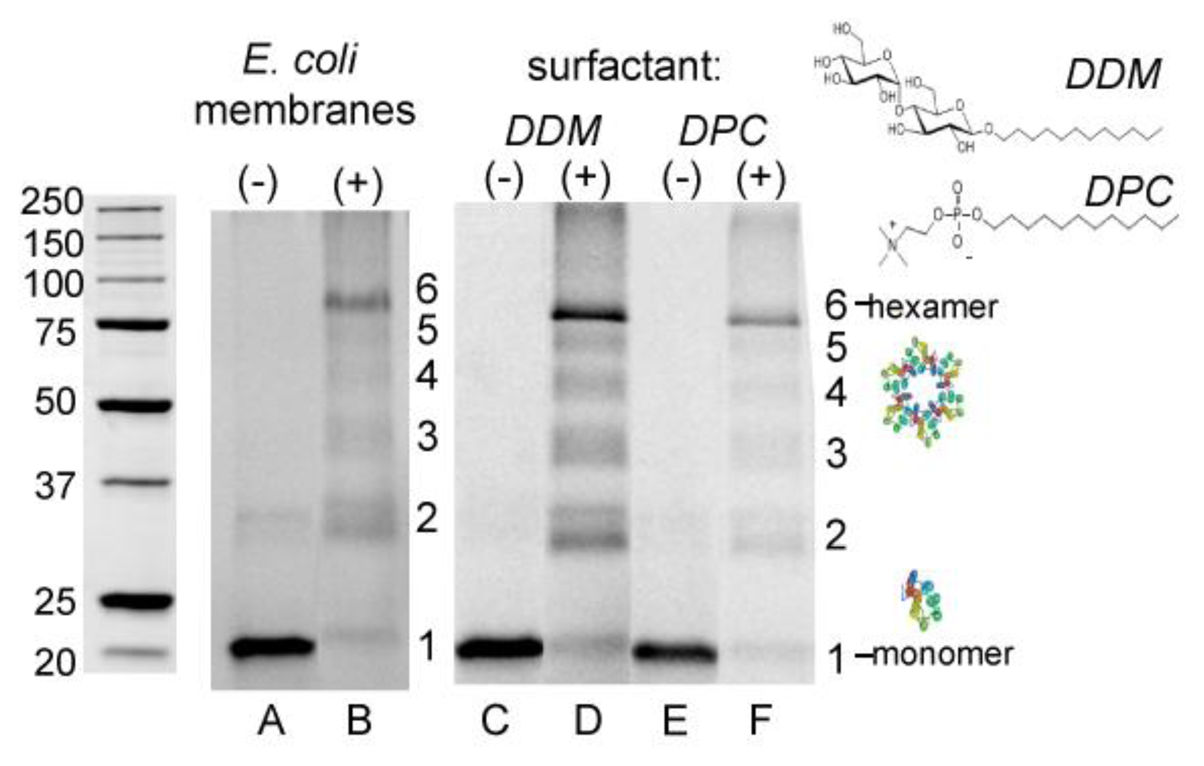
SDS-PAGE of PR in E. coli membranes with and without chemical crosslinking SDS-PAGE of untreated PR (−) and PR treated with the DSS crosslinker (+) while in extracted membranes of *E. coli* recombinantly expressing PR, using unpurified samples (A–B) and samples purified in DDM (C–D) and DPC surfactant micelles (E–F). Except for the protein marker shown (Precision Plus Standards, BioRad), all imaging was carried out with a protocol designed to specifically detect PR based on its fluorescence properties, as described in the Methods section. The doublet bands with small molecular weight differences (less than 1 kD, lanes B, D, and F: bands 2–4), can be attributed to proteolytic degradation products, as seen in other PR studies.^20^

We first sought to determine whether the oligomer observed in surfactant micelles^11^ is a physiologically relevant form, which until now has not been evaluated for a native-like bacterial membrane environment. The membrane of *E. coli* recombinantly expressing PR serves as a good approximation for the native membrane, as PR is likely oriented correctly in the complex, multicomponent bacterial membrane. When a standard polyacrylamide gel electrophoresis (SDS-PAGE) technique is used to analyze PR complexes isolated from *E. coli*, the 29 kDa PR runs as a monomeric band close to 22–23 kDa, whether in the *E. coli* membrane (Figure 1, lane A) or upon purification in surfactant micelles (Figure 1, lanes C and E). This is slightly smaller than its true molecular weight of 29 kDa, similar to what has been observed in the PR literature.^20^ Therefore, PR also displays the well-documented tendency of transmembrane proteins to migrate at rates faster than expected for their true size during SDS-PAGE, recently established to be variable due to detergent-binding and acrylamide concentration.^21; 22^ The resulting discrepancy in apparent molecular weight is caused by interactions of the protein with the anionic SDS surfactant, which can also mediate the dissolution of oligomeric contacts.^23^ In order to prevent the possibility of this and directly observe oligomeric forms of PR in *E. coli*, we supplemented the standard SDS-PAGE method with covalent chemical crosslinking.

With the aim to capture oligomers of PR present within the bacterial membrane, membranes extracted from PR-expressing *E. coli* were treated with disuccinimidyl suberate (DSS), a membrane-permeable crosslinker that reacts with primary amine groups, linking lysines that are within an ~11 Å distance (5.58–11.42 Å range according to simulation^24^). Upon SDS-PAGE analysis, we now observe the partitioning of crosslinked PR into six main bands, with the most prominent species having a much larger apparent size than the PR monomer observed without crosslinking (Figure 1, lane B). The number of bands upon crosslinking suggests that a hexameric form of PR is present, with further rationale of this stoichiometry (including purification and SEC) given in SI (section S1). As would be expected, the oligomeric structure is maintained upon purification of crosslinked PR in surfactant micelles (Figure 1, lanes D and F). The presence of lower-order constituents for crosslinked PR (bands 2–5 in Figure 1) suggests that SDS either breaks apart partially crosslinked hexamer, or that there is a distribution of oligomeric populations in the bacterial membrane. This crosslinking study convincingly shows that PR molecules are within close proximity of each other in the *E. coli* membrane, and the oligomerization of PR observed by many studies is not an artifact of reconstitution in a membrane-mimetic surfactant environment, but rather a reflection of a stable oligomeric form in the native-like bacterial membrane.

### Separation and characterization of PR-surfactant complexes

**Figure 2:**
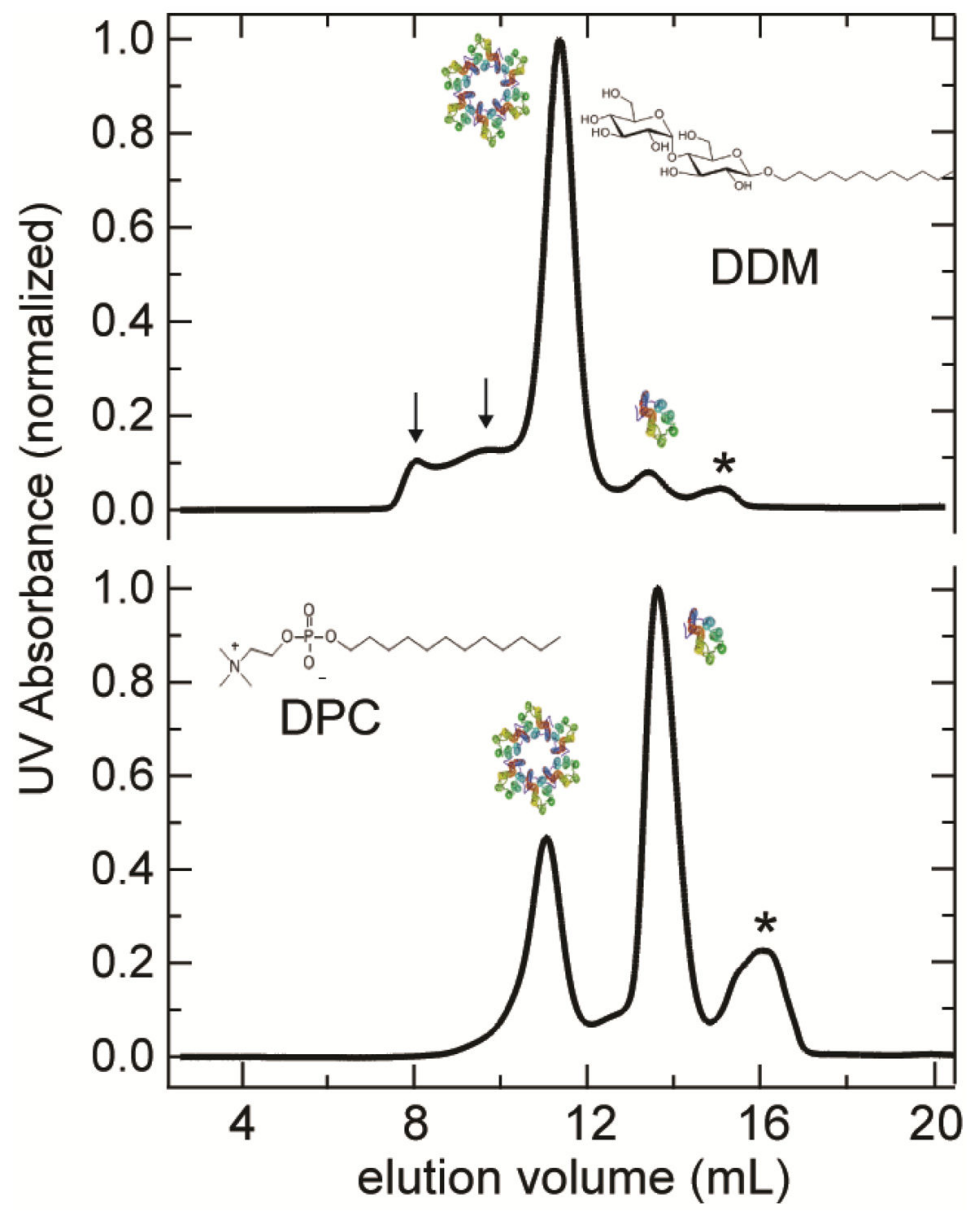
SEC separation of PR oligomers in different surfactants Characteristic SEC chromatograms of PR purified from *E. coli* and reconstituted in DDM and DPC. The distribution of oligomeric forms varies with surfactant type. Oligomeric and monomeric populations are designated as such, and have been established by light-scattering for the DDM case.^11^ Arrows indicate a higher-order aggregated species that is variable by preparation, and stars (*) indicate a blue-shifted species (maximum absorption ~380 nm). This example is of the E108Q PR mutant, but wild-type PR has similar separation patterns.

**Figure 3:**
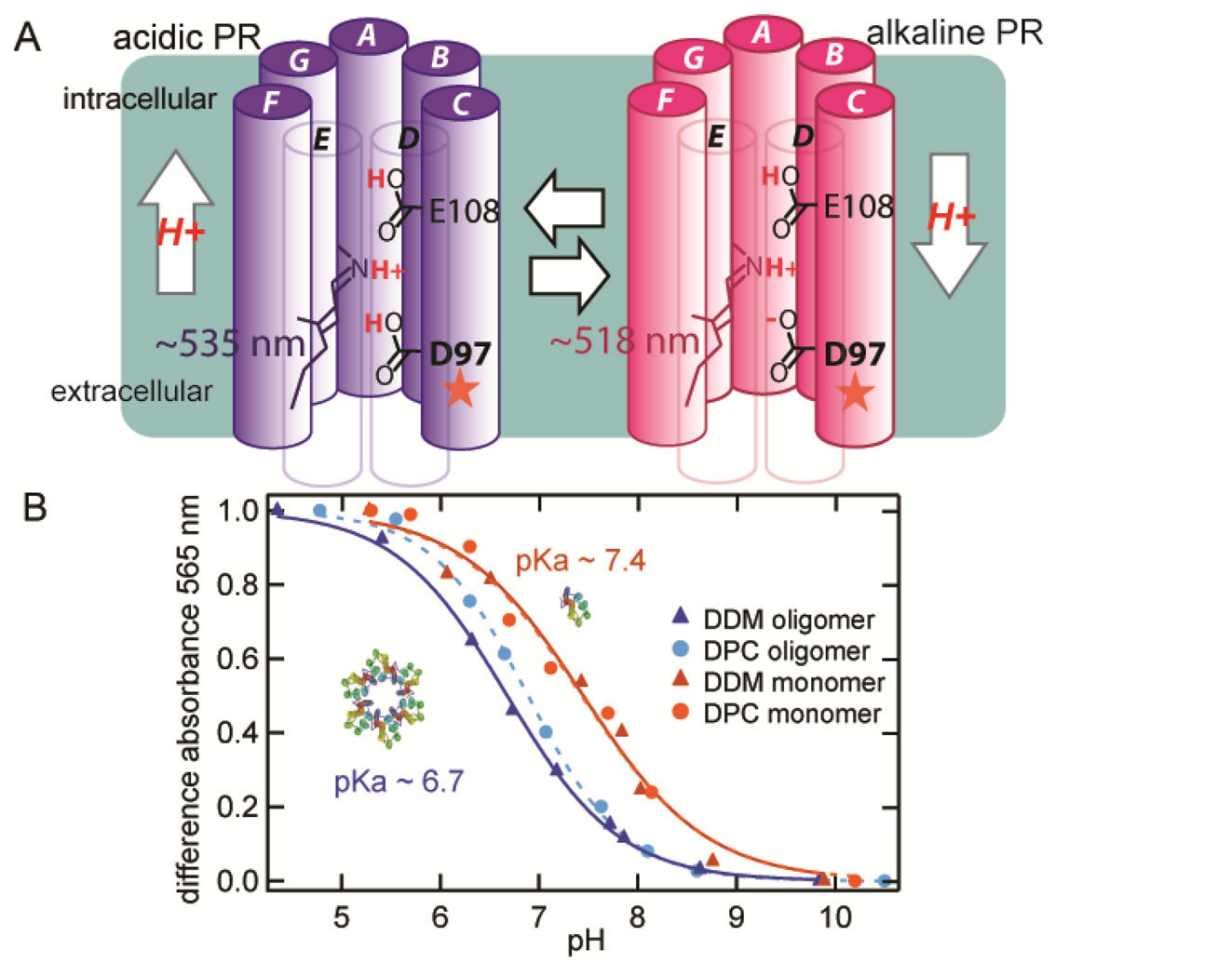
pH-dependent color transition of the PR oligomer and monomer (A) Schematic depiction of the acidic and alkaline forms of PR, which differ by the protonation state of proton acceptor residue D97, among other properties. (B) pH-dependence of the optical absorption of oligomeric and monomeric PR based on the difference spectra compared to the most alkaline spectrum (pH 9.8–10), in both DDM and DPC surfactant. The titration curves are fit to the Henderson-Hasselbach equation.

**Figure 4:**
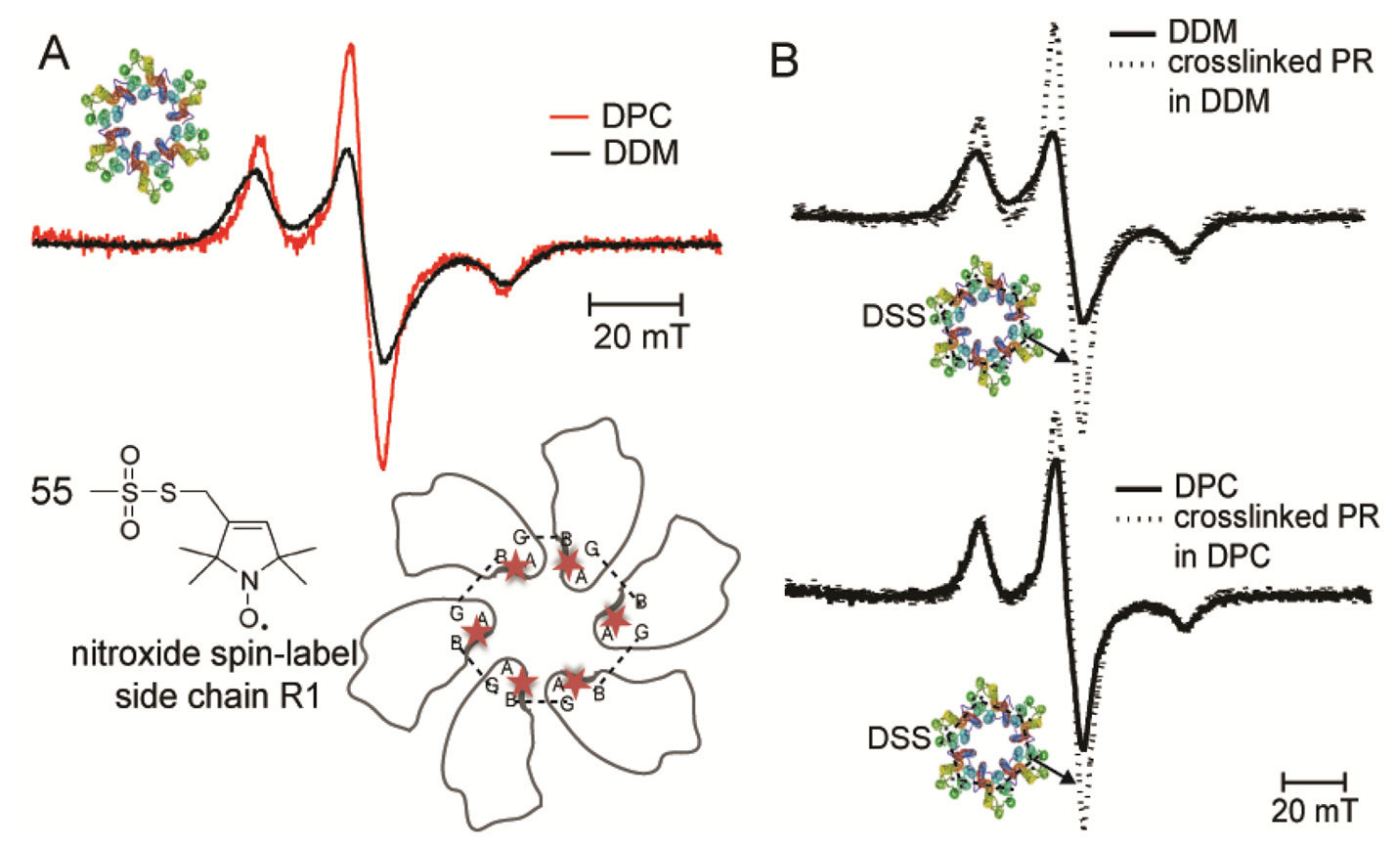
Time-resolved optical absorption of oligomeric and monomeric E108Q PR (A) Difference spectra ~80 ms after photoactivation of PR with a green light laser (t_1_) and subsequent spectra for various time points t_i_, for both the oligomer and monomer. (B) Transient buildup of the absorbance intensity at 520, indicating the decay of the M photointermediate for both the oligomeric and monomeric forms of PR in DDM. Measurements were carried out at pH 9.8. Characteristic time constants from a biexponential fit are given (τ_1_ and τ_2_).

**Figure 5:**
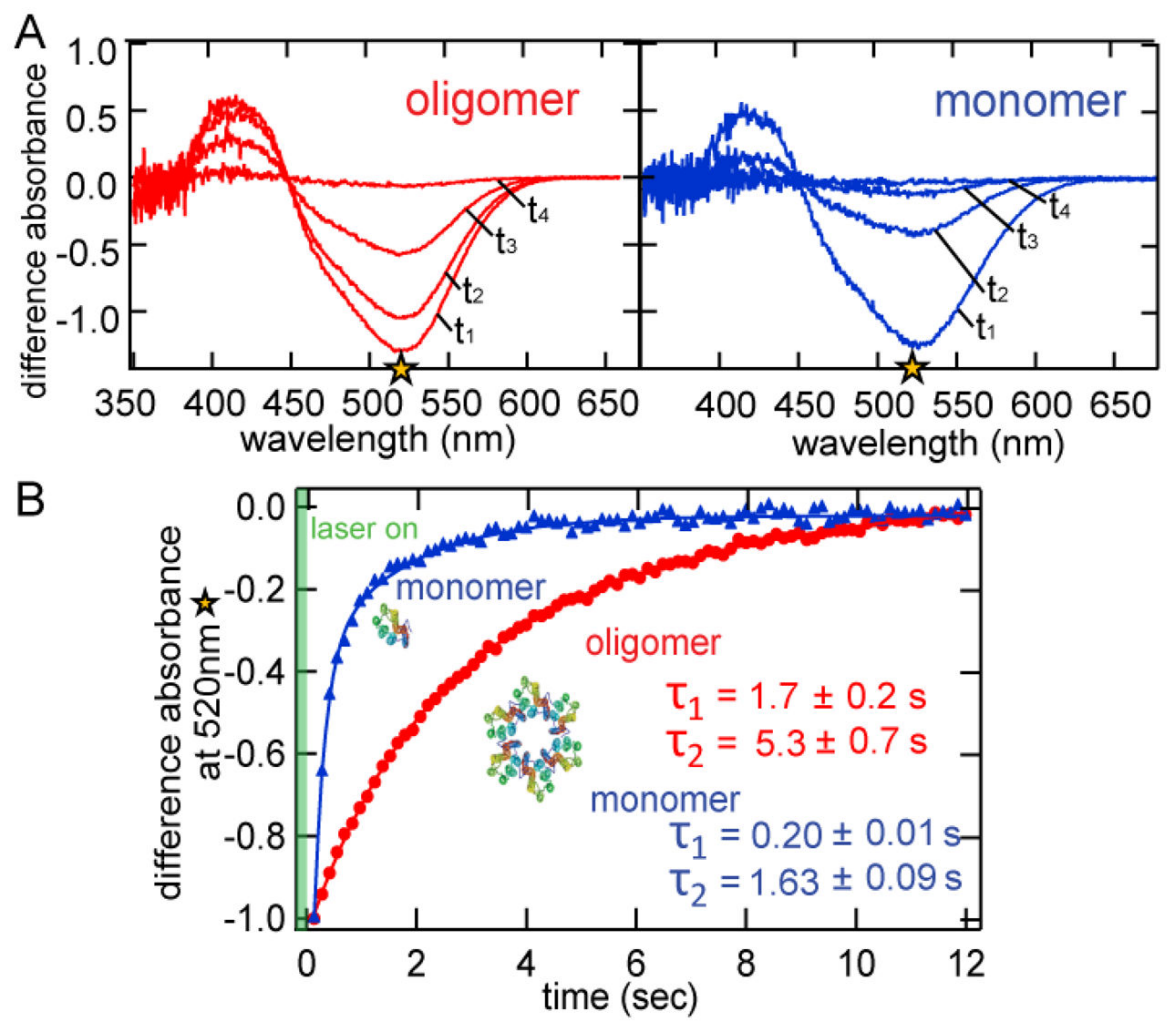
Photoactivated EPR for spin-labeled oligomeric and monomeric E108Q PR Continuous wave (cw) EPR spectra of PR spin-labeled at E–F loop site 174 before (black traces) and during (green traces) light activation by illumination with a 532 nm green laser for (A) oligomeric PR and (B) monomeric PR. The insets show time-resolved spectra taken by fixing the magnetic field to the immobile component of the spectra (denoted by a star), and observing the evolution of that spectral component during (green shading) and after green light illumination.

**Figure 6:**
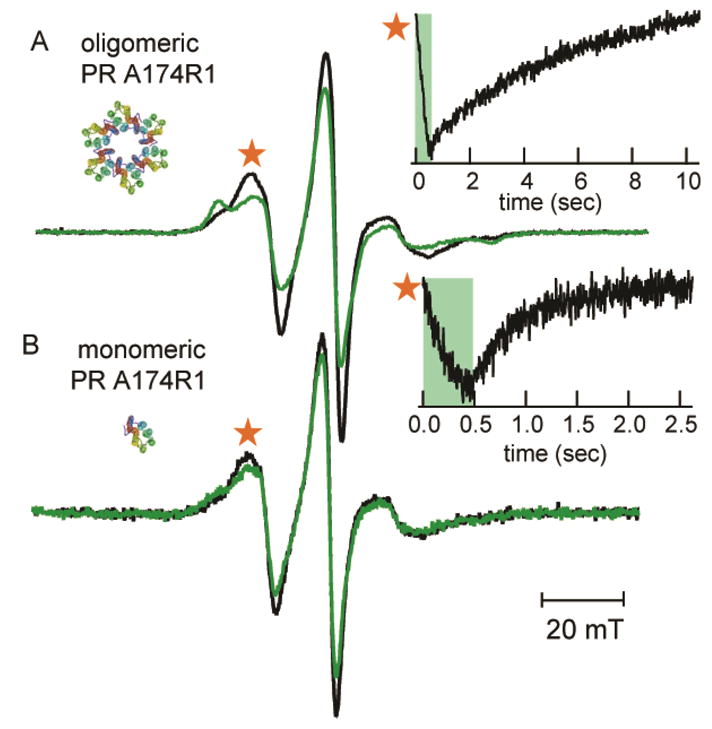
Low-temperature (140 K) cw EPR spectra of slowed-photocycle PR S55R1 in both DDM and DPC surfactants PR S55R1 (A) in the oligomeric form isolated by SEC, and (B) overlaid with DSS-crosslinked oligomeric PR. The schematic of oligomeric PR shows the spin-labeling site 55 as well as the putative crosslinking sites between protomers

The separation of oligomers within multiple surfactants enables us to isolate the functional effects of protein-protein interactions from protein-surfactant interactions, though these effects may be inherently linked within a complex cell membrane environment. By varying the surfactant used during purification and SEC, we were able to investigate the effect of surfactant on oligomer formation and its potential functional consequences. A prior SEC study has established that the DDM surfactant stabilizes oligomeric PR, which in combination with static light scattering and refractive index measurements, assigns its stoichiometry to be a predominantly hexameric state.^11^ It also resolves an oligomeric interface that was later verified by crystallography of blue-absorbing PR^14^ and further pulsed EPR analysis^15^ —which we rely on for the depiction of the hexamer given in Figures 1–6 representing the oligomeric structure of PR. We note however, that a mass spectrometry study of PR in DDM observes a pentameric form,^10^ which is also seen in crystallography of mutant versions of blue-absorbing PR^14^ and as a minority species (~10%) by AFM of PR in lipid bilayers.^13^ Taking into consideration that our experimental preparation of oligomeric PR, including SEC, is most comparable to that given in Stone, et. al,^11^ we hypothesize that we have a largely hexameric state in the oligomeric population. Nevertheless, there is a distinct possibility that pentameric PR is also present, as various studies have demonstrated it to be a stable oligomeric form. It is likely that a hexamer would not be effectively separated from a pentamer during SEC such that our “oligomer” population could in reality be a mixed state. In this study, we focus on the function-relevant aspect of PR oligomerization rather than the stoichiometry and structural properties of the PR oligomer, although we verify by SDS-PAGE and SEC that we are indeed isolating robust, multimeric PR complexes.

Here, we tested two additional surfactants: the zwitterionic DPC and the lipid-like diC7PC surfactant previously used for solution NMR characterization of PR.^25; 26^ The DDM and DPC surfactant molecules have identical hydrophobic chain lengths (12 carbons), with only the polar headgroups differing: a nonionic sugar group in DDM, and a zwitterionic phosphatidyl choline (PC) group in DPC, similar to the headgroup of a lipid molecule (structures shown in Figure 1). The choice to compare DPC- and DDM-reconstituted PR also allows us to examine how micelle geometry (size and shape) influences oligomer packing within proteo-micelles. Pure DDM micelles are oblate^21^ and range in size from 56–71 kDa,^23^ while DPC micelles are prolate^21^ and smaller, ranging in size from 18–21 kDa.^22^ Although diC7PC stabilizes PR well and corroborates the results presented here, the diC7PC data is presented in SI (Figure S2), as it does not provide a systematic comparison to pure DDM and DPC. In addition, it required us to form mixed micelles (with DDM) during SEC due to the unsustainable amount of surfactant needed, given its high cost.

The primary difference that arose from varying the surfactant used for SEC separation is that the surfactant type alters the distribution of oligomers. Comparing the SEC chromatograms of PR solubilized in the various detergents (Figure 2), it is evident that DDM stabilizes protein-protein interactions, while the zwitterionic headgroup surfactants DPC and diC7PC (which also have a propensity to form prolate micelles) enrich the monomeric form of PR, though the oligomer is still present (SEC of diC7PC shown in Figure S2). The SEC chromatograms of the eluted protein (Figure 2) show that roughly ten times more PR is in the oligomeric form than the monomer when constituted in DDM, while there is 2.5 times more monomeric PR than oligomer when constituted in DPC. There is also a smaller species indicated in Figure 2 (*) that does not have the characteristic color of PR at the pH of the separation (pH 8), rather absorbing maximally at ~380 nm. This could be a photoproduct of PR, similar to that found in other studies by mutation,^27^ or a structurally compromised variant. In any case, its quantity varies for different protein preparations, but is most easily isolated with DPC. Similarly, DPC does not maintain PR structure over long periods of time (~a week), especially at more alkaline pH and in the monomeric form, and therefore all measurements outlined in this study were carried out immediately after purification.

The oligomeric identity of the heavier PR species isolated within DPC is suggested by its elution volume, which lines up with that of the DDM population that has been found to be hexameric by static light scattering^11^ (Figure 2). Given that the chain lengths of both detergents are the same (12 carbons) and the PR oligomer is relatively large (~145–174 kD), we can assume that the relative hydrodynamic radii of the protein-detergent complexes would be dominated by the protein rather than the surfactant component. We expect this to be a good approximation as the radius of gyration of a DDM micelle has been shown by small-angle X-ray scattering to be within 2 Å of a DPC micelle.^28^ Therefore, the comparable migration of PR-detergent complexes through the column indicates that the stoichiometry of the oligomers must be similar in both cases—likely predominantly a hexamer by extrapolation of the SEC-coupled light scattering/refractive index measurement of Stone, et. al,^11^ although as stated we cannot exclude the presence of a pentameric population as found in Hoffmann, et. al., in DDM. ^10^

Nevertheless, we further implemented chemical crosslinking with DSS along with SDS-PAGE to evaluate the oligomeric state with another analytical technique. The results showed that PR crosslinked in either DPC or DDM shows a similar banding pattern (SI Figure S2). Therefore, despite drastic changes in the relative populations of the oligomeric species with the surfactant used, a structurally similar oligomeric species is retained at comparable surfactant concentrations (i.e. twice the critical micelle concentration).

### pH-dependent color transition

The primary method chosen to characterize the functionality of PR in different oligomeric states within surfactants was to observe the pH-dependence of the optical absorption spectrum. From prior studies of PR,^29^ we can consider PR to have two main pH-mediated functional forms hinging on the protonation state of a key “proton acceptor” residue in the interior of PR, D97: (1) a pink-colored alkaline conformation (absorbing maximally around 518 nm) with D97 deprotonated in the resting state, and carrying out inside-out proton transport requiring a particular “M” photointermediate, and (2) a purple-colored acidic form (absorbing maximally around 535 nm) with D97 protonated, and performing outside-in proton transport (see Figure 3A). The transition between these two functional forms is measured by taking a series of optical absorption spectra of PR under variable pH conditions, which have been found to have a single isosbestic point.^30^ The detailed description of how the pKa of D97 is determined from these measurements can be found in the SI (section S3), together with references for the prior reported pKa values for PR, which vary between 6.9 and 8.2.^29; 30; 31; 32; 33; 34^ This wide range of experimentally determined pKa values itself suggests that PR function is tuned by its environment, as different studies use a variety of surfactant types and concentrations. However, aside from mutations,^35^ the only systematic studies of D97 pKa have identified anion type and concentration^30^ as well as lipid bilayer thickness of nanodiscs^31^ as modulating factors.

Our experimental pKa values determined for PR oligomers compared to monomers demonstrate that the oligomeric form of PR in surfactant micelles profoundly affects the pH-dependent color transition, found either with optical absorption difference spectra relative to the most basic condition (Figure 3B) or the wavelength of maximal absorption (SI, Figure S3). For monomeric PR isolated by SEC, the pKa is a full pH unit (7.4–7.8) higher than for the oligomeric form (6.5–6.7) (Figure 3B). This result is found consistently for PR solubilized in either DDM or DPC surfactant, suggesting that the D97 pKa transition is affected dominantly by the protein-protein interaction conferred by the oligomeric form, and much less by varying protein-surfactant interactions.

Still, we observe one nuance of surfactant effects, where differences in surfactant headgroup change the color of PR at the more acidic pHs, notably only for monomeric PR. This is observed by a shift in the maximum wavelength of absorption from 534 nm in DPC to 541 nm in DDM (Figure S3). Though this surfactant-based color change does indicate an altered environment around the retinal chromophore, it does not appear to be functionally significant—the 5 nm shift is small considering the broadband light-absorption spectrum of PR (with typical full-width-at-half-maxima of ~110 nm), and the acidic condition is considered not physiological.

### M photointermediate as probed by the E108Q mutation

While the effect of oligomerization on the photoactive properties of PR is rigorously demonstrated by the drastic pKa shift of D97, further insight was sought by examining an important light-induced conformational state, the M photointermediate. Upon photoactivation, the M state is thought to involve the largest degree of conformational change for PR similarly to the related bacteriorhodopsin,^36^ which enables proton transport during its photocycle. The spectral signature of the M photointermediate in microbial proton pumps is deprotonation of the retinal Schiff base, which results in a significantly blue-shifted absorbance (~410 nm) compared to the equilibrium state (~520 nm), as well as all subsequent photocycle intermediates. In order to monitor the kinetics of M decay with our time-resolved optical absorption instrument, we performed these measurements on the E108Q mutant of PR, which prolongs the M state from milliseconds to seconds by inhibiting the re-protonation of the retinal Schiff base.^34^ This “slowed-photocycle” mutation has been useful for characterization studies of PR^37^ as well as for holographic applications.^38^ Crucially for this study, the E108Q mutation does not alter the distribution of oligomers isolated by SEC (Figure 2) or the trends of the pH-dependent absorption behavior of the PR monomer or oligomer (SI, Figure S4).

The evolution of the M state for PR oligomers was observed by time-resolved optical absorption measurements, as well as with time-resolved and site-specific EPR of spectral signatures that were previously identified to signify conformational changes of an E–F loop site.^37^ The optical absorption difference spectra obtained after illumination of the E108Q mutant of PR with a green (532 nm) laser are shown in Figure 4A. An increase in a population corresponding to the M photointermediate is evident by the positive difference absorption at 410 nm, as is a decrease of the equilibrium PR state, indicated by the negative difference absorption at 520 nm. This spectral feature is observed for both the PR oligomer and the monomer constituted in DDM indicating that the M state structural conformation is a common and characteristic feature of PR, regardless of its oligomeric state. However, when the kinetics of the transient decay of the M photointermediate is measured by the repopulation of the equilibrium PR state at 520 nm, we observe a biexponential decay with characteristic time constants that are roughly five times longer for oligomeric PR compared to the monomer (Figure 4B). Similar trends upon changing oligomeric state are found for the other surfactants tested, DPC, and diC7PC, although the absolute values of the kinetic time constants obtained vary with surfactant type (SI, Figure S5), while monomeric PR in DPC proved to be unstable at the alkaline pH of the time-resolved measurement (pH 9.8). This analysis reveals a prominent influence of oligomerization on the kinetics of M state decay for the E108Q mutant, with inter-PR contacts slowing it down significantly.

In order to complement the time-resolved optical absorption measurements tracking the decay of the M photointermediate of “slowed-photocycle” PR E08Q, we conducted continuous wave (cw) EPR measurements on the same PR spin-labeled at an E–F loop residue. EPR provides a site-specific technique to probe nanosecond-scale dynamics from the perspective of nitroxide spin probes that are covalently attached to the protein by reaction with a single cysteine that is introduced at a residue of interest by site-directed mutagenesis. Changes in local dynamics due to variable interactions with the surrounding protein surface and water, for example from residue-to-residue variation or an altered protein conformation, result in different EPR spectral signatures.

The spin-labeled 174 side chain chosen is known from our prior work ^37^ to experience a large degree of light-driven conformational change, namely an immobilization of the side chain by interaction with the protein surface. This is manifested by a decrease in a characteristic mobile component of the EPR spectrum (denoted as a star in Figure 5) upon illumination with a 532 nm green laser light, with a concurrent increase in the characteristic immobile component. We see the same qualitative behavior for PR isolated in its oligomeric form in DDM (Figure 5A), only more pronounced than in the previous study, where a mixture of oligomeric and monomeric PR in DDM was used ^37^. For monomeric PR, there was also a decrease in the mobile spectral feature, however to a much lesser degree compared to that seen in oligomeric PR (Figure 5B). Transient tracking of this immobile component revealed that the quality of conformational change (immobilization upon photoactivation of PR) is preserved for both oligomeric and monomeric PR, but the M state decays on a much faster timescale for the monomer, in agreement with the optical absorption measurement observing faster conformational changes for monomeric PR (Figure 4). Therefore the reason for the less apparent spectral change for the monomer (Figure 5B) is not because monomeric PR does not undergo the same light-driven conformational change as observed for oligomeric PR, but rather because the photoactivated state is shorter-lived and thus its spectral signature cannot build up adequately during the measurement, which is possible for the oligomeric form of PR due to its relatively slow photocycle kinetics. This agreement of the optical absorption (Figure 4) with EPR firmly verifies that the local conformational transformation of the E-F loop, as tracked by time-resolved EPR, is a representative component of PR’s M state decay as tracked by time-resolved optical absorption, as has been postulated before.^37^

### Altered packing of the oligomer by surfactant environment and crosslinking

Having observed the prominent role of oligomerization in tuning the key functional properties of pH-dependent light absorption, we sought to explore the physical quality of surface packing of PR molecules within the oligomer. The crystal structure of blue PR, as well as sparse distance measurements of green PR, reveal specific protein-protein interactions and orientation to underlie PR oligomers. From continuous wave (cw) EPR lineshape analysis, it has been shown that site S55 lies within roughly 16 Å of the same residue of an adjacent PR in the hexamer in DDM, such that the EPR spectrum is broadened due to the distance-dependent dipolar interaction between nitroxide spin-labels (side-chain R1 shown in Figure 6) at the 55 site.^11^

We investigated the effect of surfactant environment on this dipolar broadening to gain insight into the distance between PR molecules by measuring low-temperature cw EPR lineshapes of oligomeric PR spin-labeled at the 55 residue (Figure 6A). The most apparent feature of the low-temperature cw EPR is the much greater intensity of the spectrum of PR S55R1 in DPC (Figure 6A), with both spectra normalized to spin concentration, signifying less broadening and therefore a less tightly packed oligomer than in DDM. In fact, the apparent loss of dipolar broadening indicates that the distance between adjacent A–B loop 55 residues is greater than 20 Å when oligomeric PR is reconstituted in a DPC micelle. These dipolar broadening effects are also observable with room-temperature EPR spectra (SI, Figure S6). We note that the S55C mutation and subsequent spin labeling (S55R1) did not affect either the distribution and isolation of oligomeric states, nor the general trends observed for the key oligomer-dependent functional properties (SI, Figure S8). It is insightful that these properties, including the D97 pKa and photocycle kinetics, are upheld in either the DPC or the DDM micelle environment (Figures 2 and 3), while the packing of PR in the oligomers significantly vary (Figure 6A).

Crosslinking PR within the *E. coli* membrane with the lysine-reactive DSS (~11.4 Å) captures the oligomer, as demonstrated by SDS-PAGE (Figure 1). Therefore the isolation of crosslinked protein upon subsequent purification and SEC can evaluate any functional changes upon permanently forging some oligomeric contacts. The possible sites that can be connected by crosslinks between PR molecules in the hexamer (as delineated in Figure 6) were found using the solvent-accessible distances (see SI, section S6).^39^ Low-temperature cw EPR lineshape analysis demonstrates that the distance across the oligomer measured at the A–B loop 55 residue is extended by DSS crosslinking in both DDM and DPC environments (Figure 5B), as indicated by the loss of dipolar broadening (see higher intensity of the center peak upon normalization to spin concentration). Again, despite the obvious changes in the physical packing of PR within the oligomer, the pH-dependent optical absorption measurements reveal only small changes upon crosslinking (SI, Figure S7), with the pKa transition of ~7.0 for the PR oligomer upheld but shifted by 0.02–0.4 pH points, depending on the analysis method.

## DISCUSSION

### PR forms oligomers in bacterial membrane and surfactant micelle environments

The presence of a oligomeric structure of PR within the *E. coli* membrane verified by SDS-PAGE and crosslinking represents the first finding suggesting a propensity of PR to make homo-oligomeric contacts in a bacterial lipid bilayer environment (Figure 1). Aside from the six bands visible on the gel upon crosslinking, further evidence for a particular hexameric form is given by SEC of purified crosslinked PR (SI, Figure S1), coupled with light-scattering.^11^ We must note that PR could be overexpressed and the precise *E. coli* lipid composition differs from the native organism, which could affect oligomerization. Nevertheless, these factors can be regulated by the organism, and therefore the PR oligomers observed^11,14^ emerge as physiologically important forms for which the functional influence should be investigated. Our results also indicate that the noncovalent interactions holding the oligomer together are relatively weak, as evidenced by the dissolution of oligomeric contacts upon exposure to SDS during SDS-PAGE, and also the distribution of various oligomeric forms including monomers even after crosslinking to capture the oligomer (Figure 1).

Upon isolation of the oligomeric and monomeric species within micelles for characterization, it becomes clear that surfactant properties affect the distribution of oligomers (Figure 2). The tendency of DPC to isolate monomeric PR is possibly facilitated by the prolate shape of DPC micelles, which would be disrupted upon reconstitution of PR oligomers, which have a larger width than height—an oblate-type structure that is therefore more readily stabilized by the oblate shaped DDM micelle. These hypotheses are corroborated by SEC on PR solubilized in diC7PC (SI, Figure S2), which behaves similarly to DPC in terms of enriching the PR monomer, likely due to its identical headgroup and similar micelle size and shape. However, PR proves to be more stable in diC7PC, retaining its characteristic color for months or longer, suggesting that the short double-chain is more advantageous for enabling PR function than the twelve-carbon single chain of DPC.

### PR function is tuned by oligomeric contacts

In spite of the potentially weak nature of the interactions holding together the PR oligomer, the studies presented here suggest the specificity of the oligomeric interface exerts a profound functional influence, which we find to dominate over protein-surfactant effects for tuning transmembrane protein function. This is epitomized by the dramatic (1 pH point) shift for the pH-dependent protonation transition of the interior D97 residue upon removing the interaction mediated by oligomerization (Figure 3). Looking to understand this phenomenon further, the relatively high pKa of D97 compared to the corresponding value for bacteriorhodopsin’s primary proton acceptor (D85), 2.6,^40^ has been attributed to its interaction with H75,^41; 42^ thought to be a conserved feature of eubacterial proton pumps as distinguished from archaeal variants.^41^ It is notable that this residue of the B helix is close to the oligomeric interface, and indeed the crystal structure of the homologous blue-absorbing PR resolves a hydrogen bond between H75 and D97, as well as with W34 of an adjacent PR within the hexamer.^14^ Our observed pKa changes for D97 in green PR upon oligomerization corroborate the existence of a specific oligomeric interface as resolved in the crystal structure. Monomeric PR would neither have the cross-protomer W34-H75 interaction nor other oligomeric contacts, such that the H75-mediated modulation of the D97 proton acceptor’s protonation state would differ from the oligomer, and result in a higher pKa (Figure 2).

The wide range in pKa values for PR found in the literature^32; 33; 34^ is consistent with sample-to-sample variation in oligomeric populations unless the oligomeric states are separated before characterization. pKa values determined in similar methods to ours range from 6.9 to 8.2 in a variety of surfactant environments,^31^ including studies of PR expressed in lysed *E. coli* membranes,^32^ solubilized in various concentrations of the octylglucoside (OG)^33^ and dodecylmaltoside (DDM) nonionic detergents,^30; 34^ and reconstituted in phospholipid nanodiscs^31^ and liposomes.^29^ Two of our results presented here can account for this wide range—(1) the surfactant type alters the distribution of oligomeric forms (Figure 2 and Figure S2) and (2) pKa is significantly dependent on oligomeric state (Figure 3 and Figure S3). In studies that use higher surfactant concentrations, higher pKa values are obtained (e.g. 8.2 for 3 wt% octylglucoside (OG)^33^), corresponding to a greater contribution of the monomer population. The effect of oligomeric contacts on D97 pKa could also explain the reported observation in Partha, et. al,^33^ that the measured pKa in OG decreases as the PR sample ages over months, presumably as a monomer-enriched population (pKa~8.2) converts into more stable oligomeric species (pKa~7.2). Similarly, DDM stabilizes the oligomer and therefore results in values only slightly higher than the oligomeric pKa found here (7.0–7.1) potentially due to the presence of some monomeric species, even for higher concentrations of surfactant (0.2^34^–0.5^32^ wt%).

Finally, our observation of a distinct pKa shift of D97 upon oligomerization of PR suggests the potential for altered activity at the pH of the native ocean environment (~8.2) based on oligomeric contacts. The pKa differences observed would mean that protonation of the key “proton acceptor” residue (D97) is altered from >90% in the oligomer to ~50% for the monomer, suggesting that the population of PR with a particular internal state is dramatically changed by intra-oligomer contacts. Since the oligomeric form of PR could be varied by either expression levels or lipid composition, it would be a viable way for the cell to regulate the function of PR. We note, however, that the studies presented here are in a micellar environment, and the additional impact of the lipid bilayer has yet to be considered.

The additional time-resolved measurement of M photointermediate decay facilitated by the E108Q mutation provides further evidence for the significant role of oligomerization in tuning PR function, namely slowed M decay for the oligomeric form (Figures 4 and 5). The qualitative observation of preserved yet slowed M state-related conformational change for oligomeric PR is also corroborated by EPR measurements (Figure 5), where an E–F loop side chain experiences decreased mobility upon light-activation but decays back to equilibrium more slowly when in a oligomeric form. Since many other seven-helical transmembrane proteins have been shown to also go through a M-like intermediate while carrying out their function, including sensory proteins^43; 44^ and even mammalian receptors,^45^ it represents a significant functional state. The altered kinetics of light-induced conformational change suggests that the structural differences due to the specific protein-protein contact inherent to oligomerization stabilize the M photointermediate. These measurements were taken at an appropriately alkaline pH (9.8) chosen from the D97 pKa curves of E108Q PR (Figure S4), such that the relative populations of the deprotonated PR form would be similar for both the oligomer and the monomer. While the physiological utility of altered timescales of protein activity is not obvious for PR, it does provide an interesting functional consequence of oligomerization that contributes to the discussion of microbial rhodopsin function, which is becoming an increasingly diversified subject, due in part to recent discoveries of alternative signal transduction mechanisms such as in *Anabaena* sensory rhodopsin^43^ which also form an oligomeric structure.^46^

### The surfactant environment preserves functional properties but influences oligomeric contact

The functional measurements presented in Figures 3, 4, and 5 point to a dominant influence of oligomerization over surfactant environment, as it is evident that any changes due to surfactant type (DDM vs. DPC) are minor in comparison. If we were to attribute the prominent pKa modulation upon PR oligomerization observed here (Figure 3) to a specific interaction, it would be the cross-protomer interaction between residues W34 and H75 of an adjacent PR revealed by the crystal structure (a strong hydrogen bond of less than 3 Å).^14^ It would be expected that the large displacement observed when going from DDM to DPC (~4 Å or more, Figure 6) would disrupt this interaction, yet it is evident that the pKa is not greatly affected by changing the surfactant from DDM to DPC (Figure 3), and only the complete dissolution of oligomeric contacts immensely alters this functional property. Aside from the untested possibility that the specific cross-protomer W34-H75 interaction is not the sole factor accounting for the pKa of PR, this suggests that the protein structure may deform in such a way as to enable the W34-H75 hydrogen bond to be preserved between adjacent PRs upon reconstitution in DPC, or that the micellar environment is sufficiently flexible or dynamic to allow this interaction even with such loose packing.

Similarly to altering the surfactant from DDM to DPC, crosslinking extends the distance between neighboring 55 residues, as observed by cw EPR, to result in a more loosely-packed oligomer (Figure 5B). As for functional consequences, crosslinking yielded a greater pKa shift than that observed from surfactant changes (0.02–0.4 pH points), and slightly slowed M decay (Figure S9). Nevertheless, these key functional properties are not affected as much as they are by the oligomerization of PR (Figures 3–4). The inherent DSS crosslinker flexibility, also indicated by the ~6 Å distance variability found from simulations,^24^ could be the key parameter enabling oligomeric contacts in spite of a greater equilibrium distance between PR molecules within the oligomer. The monomer state would abolish this interaction entirely, in contrast to oligomeric forms where adjacent PR molecules are spread apart by surfactant or by flexible crosslinkers.

## CONCLUSION

The spectroscopic studies of PR presented here clearly demonstrate a function tuning role of transmembrane protein oligomerization, which could be governed *in vivo* by cellular regulation of lipid composition, expression levels, or both. This was observed by a prominent (1 full pH point) decrease in pKa of a functionally important internal residue of PR (D97) (Figure 3) and a fivefold slower M state decay for the oligomeric vs. the monomeric state of PR in surfactant micelles (Figures 4 and 5). The molecular-level detail regarding the PR oligomer-micelle complex obtained by EPR reveals that the packing of molecules within a given surfactant environment can be altered (Figure 6) with negligible functional consequences. It could be the dynamic nature of the complex that nevertheless allows specific interactions to take place between adjacent proteins, for example the cross-protomer W34-H75 interaction.^14^ The fluid cell membrane facilitates diffusion of transmembrane proteins in a two-dimensional matrix and thus can promote protein-protein interactions. Therefore even weak interactions between transmembrane proteins could potentially have potent functional consequences. Although it remains to be seen whether the oligomeric state directly tunes function within the native organism expressing PR, the clear functional influence of oligomerization exemplifies a versatile function-tuning mechanism of transmembrane proteins that likely has implications for multimeric protein systems beyond the PR oligomer.

## MATERIALS AND METHODS

The expression and purification of cysteine-free green-absorbing PR, both with and without the E108Q mutation that enriches the M state of PR, was largely carried out similarly to previous studies.^11; 37^ SEC chromatography to isolate the different oligomeric forms of PR was conducted as described in Stone, et. al,^11^ however using buffer with the DPC and diC7PC surfactants in addition to DDM. Crosslinking and SDS-PAGE were performed according to standard procedures, with details given in SI.

The pH-dependent optical absorption spectra of wild-type PR and the variants studied here were measured with a Shimadzu UV-1800 spectrophotometer. A home-built time-resolved optical absorption spectrometer, as described in a prior study and further in SI,^37^ was used to observe the photocycle kinetics after photoactivation of the slowed-photocycle E108Q mutant with a 532 nm green laser. Snapshots of the difference spectrum upon activation were taken ~80 ms apart in the visible range (350 nm to 700 nm). The slowed-photocycle S55C PR mutant was spin-labeled as in a prior study,^11^ and studied with X-band (0.35 T) EPR measurements at room temperature and low temperature (150 K), with spectrometer details and spectral parameters given in SI.

## Supplementary Material

supplement

## HIGHLIGHTS

- Functional implications of transmembrane protein oligomers are not well understood
- Crosslinking establishes that PR oligomers are present in the bacterial membrane
- Oligomers and monomers of Proteorhodopsin (PR) are isolated in surfactant micelles
- Specific oligomeric interactions tune measurable light-driven PR activity
- Oligomerization is established as a key determinant of PR function

## ABBREVIATIONS

PR: proteorhodopsin
EPR: electron paramagnetic resonance
7TM: seven-transmembrane
DDM: n-dodecyl-β-D-maltoside
DPC: n-dodecyl-phosphocholine
SEC: size exclusion chromatography
FPLC: fast protein liquid chromatography
SDS-PAGE: sodium dodecyl sulfate-polyacrylamide gel electrophoresis
DSS: disuccinimidyl suberate
