# Supplementary material for: Functional consequences of the oligomeric assembly of Proteorhodopsin
Source: J Mol Biol. Author manuscript; Available in PMC 2016 Mar 27. (PMC4374980; doi:10.1016/j.jmb.2015.01.004)
Supplement: supplement [file NIHMS662283-supplement.docx]

SUPPLEMENTAL INFORMATION

**(S1) Analysis of PR oligomeric states within extracted *E. coli* membranes upon crosslinking**

For the PR sample crosslinked within the *E. coli* membrane, six bands are visible on the SDS-PAGE gel (Figure 1, lane B), which suggests that a specifically hexameric oligomeric structure of PR is stabilized by crosslinking. Though the apparent size of the hexameric complex (~81 kD) is significantly smaller than the weight corresponding to six-fold the monomer band, this can be attributed to migration differences between the hexamer and monomer, documented for various others transmembrane oligomeric complexes.^1^ Further evidence suggesting that a hexamer is a significant species trapped by crosslinking and visualized in the gel is that upon purification of PR crosslinked within the bacterial membrane and reconstitution in n-dodecyl-β-D-maltoside (DDM) (which does not change the banding pattern on the gel, main text, Figure 1, lane C), the size-exclusion (SEC) chromatogram retains similarly sized complexes as PR isolated in DDM without prior crosslinking (Figure S1). As static light-scattering has established that the predominant oligomeric complex of PR in DDM separated by SEC is a hexamer,^2^ this implies that the crosslinked species extracted from the membrane is likely of the same form. We note here (as in the main text) that our data does not exclude the concurrent presence of a pentameric form of PR within the membrane or in detergent, although we hypothesize that the hexamer is predominant by extrapolation of the conclusions given in Stone, et. al^2^ in addition to our crosslinking/SEC data.

**Figure S1.** SEC chromatograms of wild-type PR purified from *E. coli* and reconstituted in DDM surfactant. The same oligomeric forms are visible with and without DSS crosslinking within the extracted membrane, including a hexameric species established from a prior static light scattering study.^2^ Oligomeric and monomeric populations are designated as such, while arrows indicate a higher-order aggregated species, and the star (*) indicates a blue-shifted species.

**
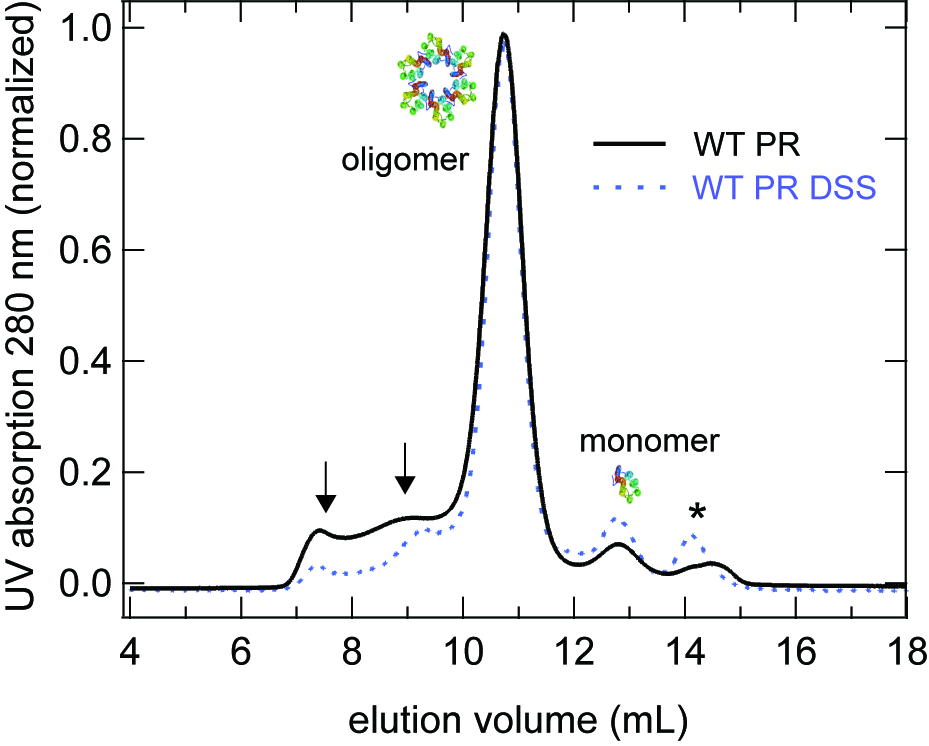
**

**(S2) Capturing the oligomeric state of PR within different surfactant environments**

Here, we extended work done by a previous study^2^ to apply size-exclusion chromatography to PR in three detergent environments: the nonionic DDM, zwitterionic DPC, and the lipid-like diC7PC. DPC was chosen as a counterpart to DDM not only to examine the headgroup effect on PR oligomer stabilization or function, but also any changes in the protein-detergent complex that could be incurred from its different micellar properties, namely the prolate (vs. an oblate DDM) geometry^3^ and smaller size (~18-21 kDa for DPC^4^ vs. 56-71 kDa for DDM^5^). The low CMC (0.18 mM^5^) of DDM makes it an economically advantageous choice compared to DPC (1.1 mM^5^), while the large micelle size of DDM often maintains robust protein activity.^6; 7^ However, this same quality can make DDM unsuitable for solution NMR structural studies as it forms large, slow-tumbling, protein-detergent complexes.^6^ DPC, in contrast, has been successful for ~40% of all NMR membrane protein structure determination studies, likely owing to its property of forming smallest and dynamic protein-detergent complexes.^8^ diC7PC likely forms similarly small, prolate micelles by analogy to other short-chain phospholipids^9^ at a CMC similar to DPC (1.1-1.4 mM).^7^

When the zwitterionic diC7PC surfactant was employed for SEC separation, the protocol was modified to minimize the amount of diC7PC used by incubating the protein in a high concentration of diC7PC (2 wt%) before separation via SEC for which a DDM-containing buffer was used. This procedure (Figure S2A) significantly increased the population of monomer over a procedure using only DDM surfactants (main text, Figure 2), simultaneously demonstrating (1) the propensity of diC7PC to stabilize the PR monomer, accounting for its successful use in NMR structural determination,^10^ and (2) the weak nature of the oligomeric interaction, such that it is not readily reformed upon contact with the excess of DDM that is known to stabilize hexameric PR. Unlike DPC, however, both hexameric and monomeric PR isolated in such a fashion have good long-term structural stabilities, even when buffer-exchanged and stored in diC7PC, suggesting that the seven-carbon double chain of diC7PC is advantageous over the twelve-carbon single chain of DPC.

The hexameric identity of the heavier PR species isolated within DPC (denoted in main text, Figure 2) can be deduced from its elution volume, which lines up with the DDM population that has been found to be predominantly hexameric by static light scattering.^2^ Therefore, we implemented chemical crosslinking with DSS along with SDS-PAGE to firmly evaluate the oligomeric state in the two surfactants. As evident from the gel electrophoresis, PR crosslinked in DPC and DDM after purification shows a similar banding pattern (lanes 2 in Figure S2B), suggesting that despite the changes in the population of the oligomeric species with the surfactant used, its identity is retained. The PR aggregate pointed out by arrows in Figure S2 has been identified as such by light scattering, further established by SDS-PAGE analysis by comparing to PR that is crosslinked while still in the *E. coli* membrane and then purified in
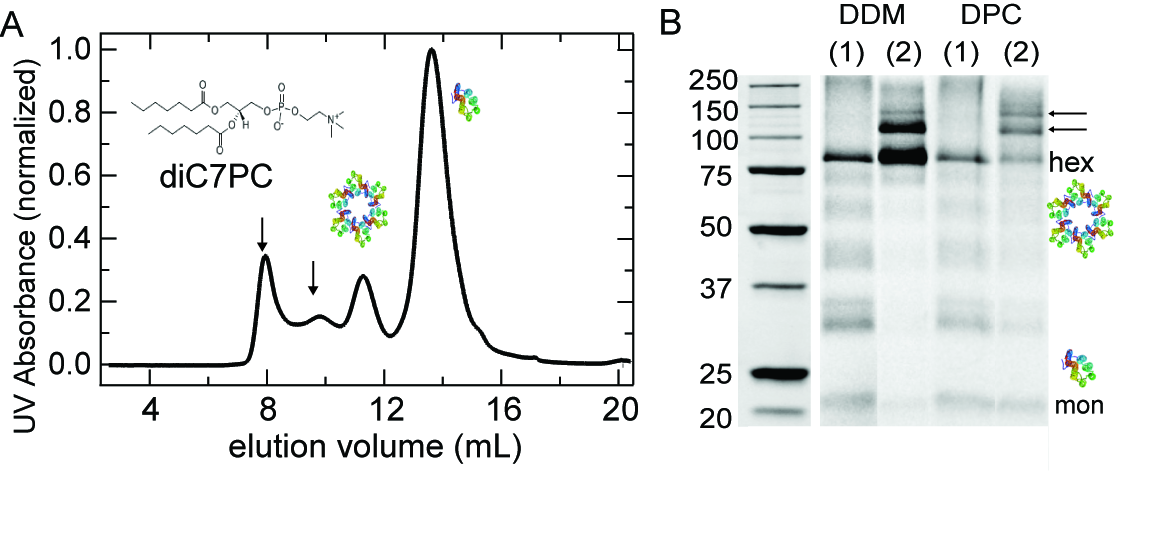
the appropriate surfactant (lanes 1 in Figure S2B). We expect that the largest species obtained from crosslinking in the membrane (denoted “hex” in Figure S2B) is the physiologically relevant form, and indeed the amount of “aggregate” does vary from one preparation to another.

**Figure S2.** (A) SEC chromatograms of PR purified from *E. coli* and reconstituted in diC7PC. DDM was used as the SEC running buffer after overnight incubation in a large amount of diC7PC (2 wt%). (B) SDS-PAGE of PR crosslinked with DSS and reconstituted in DDM or DPC surfactant. Samples in lane (1) were crosslinked in the membrane and then purified, while PR in lane (2) was crosslinked after purification in surfactant. In both a putative hexameric form is present, although post-purification (lane 2) higher-order aggregates are more evident, indicated by arrows.

**(S3) Determining the pH-dependent absorption behavior of PR (D97 pKa)**


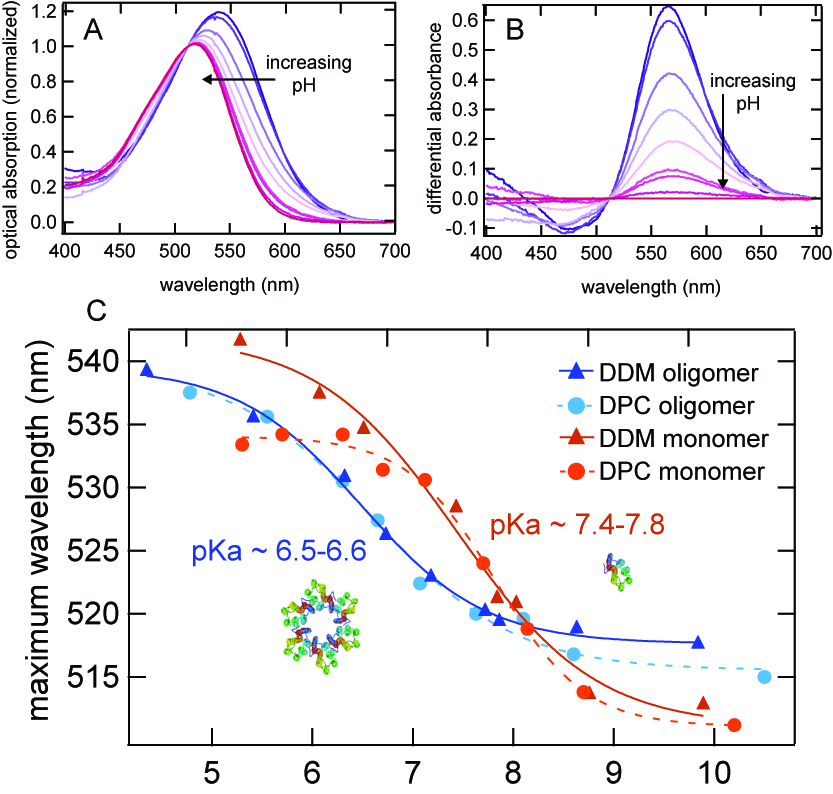
Two methods are widely used in the PR literature to determine the pH-dependent optical absorption transition, which is related to the pKa of the D97 “proton acceptor” residue. Both methods require a series of optical absorption spectra measured for PR under different pH conditions, which results in a single isosbestic point (shown in Figure S3B). The first method to evaluate pKa, an example of which is given in Figure S3C, is to simply plot the pH-dependence of the maximum wavelength of the absorption spectra.^11^ This results in a logarithmic curve that can be fit to the Henderson-Hasselbalch equation to obtain the pKa of the color transition (equation given in section S9). A second type of analysis is based on the pH-variant difference spectra.^12^ This involves subtracting each absorption spectrum from the most basic one, which results in a difference spectrum that has a maximum close to 565 nm (shown in Figure S3C). The amplitude of the differential absorption at this maximum can be plotted against the pH condition (Figure S3A) to similarly result in a logarithmic curve that can be fit to obtain the D97 pKa, with our key result being that the PR monomer has a significantly higher pKa (7.4) than the oligomer (6.7), shown in main text, Figure 3. We find that the two methods give pKa values within error of each other, although the cooperativity parameter (*n*) can differ, which still range from 0.6-1 for all samples measured, similar to in other studies of PR. Therefore we can characterize the protonation of D97 as having a negative cooperative behavior.

**Figure S3.** (A) Example optical absorption spectra of PR upon varying the pH (shown for the oligomer in DDM) and (B) difference spectra when the most alkaline spectrum is subtracted. (C) pH-dependence of the optical absorption of PR based on the maximum wavelength of absorption, in both DDM and DPC surfactants.

**(S4) pKa measurement for the slowed-photocycle mutant PR E108Q**


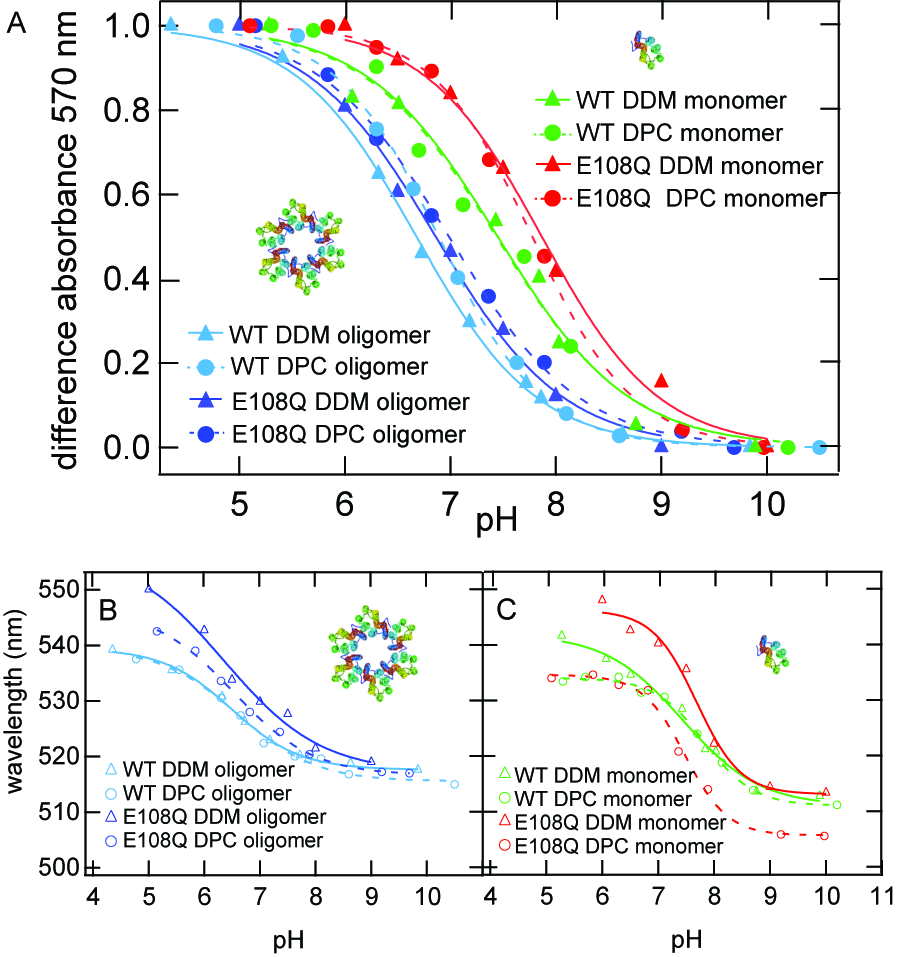
Considering the various factors that could influence the internal properties of the channel, we also sought to evaluate whether the E108Q mutation altered either the pH-dependent absorption behavior of PR, particularly the drastic effect of oligomerization observed for wild-type PR (main text, Figure 2). We find that the D97 pKa of both the oligomeric and monomeric forms of the slowed photocycle PR are similar to that found for wild-type PR, in either the DDM or DPC surfactant (Figure S4), and the trend of a significant red-shifted pKa (~1 pH point) for monomeric PR preserved. Interestingly, the color change for slowed photocycle PR is more pronounced when surfactant is varied compared to wild-type PR, with a 10 nm shift for monomeric PR in DDM relative to DPC at all pH conditions (Figure S4C). There are also distinctive changes to the shape of the pKa curves corresponding to the oligomeric state, resulting from a change in the cooperativity parameter from 0.7 for the oligomer to 1 for the monomer (Figure S4). In addition, the E108Q mutation seems to affect the affect the pKa of the monomer more so than the oligomer (slight shift from ~7.5 to ~7.9).

**Figure S4.** (A) pH-dependence of the optical absorption of oligomeric and monomeric PR based on the difference spectra compared to the most alkaline spectrum (pH 9.8-10), in both DDM and DPC surfactant, for WT PR and “slowed-photocycle” mutant E108Q. The pH-dependence of the optical absorption of WT and PR E108Q based on the maximum wavelength of absorption, for the oligomeric form (B), and the monomeric form (C).

**Table S1.** pKa values for WT PR and PR E108Q**,** as found from difference spectra analysis (shown in Figure S4A) and fits to the Henderson-Hasselbalch equation (Equation S2, indicated below in section S9)

| oligomeric state | detergent | mutant | pKa | n |
| --- | --- | --- | --- | --- |
| Oligomer | **DDM** | WT | 6.68 ± 0.02 | 0.76 ± 0.02 |
|  |  | E108Q | 6.86 ± 0.04 | 0.72 ± 0.05 |
|  | **DPC** | WT | 6.89 ± 0.02 | 0.88 ± 0.04 |
|  |  | E108Q | 6.99 ± 0.04 | 0.71 ± 0.04 |
| Monomer | **DDM** | WT | 7.45 ± 0.07 | 0.70 ± 0.08 |
|  |  | E108Q | 7.87 ± 0.04 | 0.78 ± 0.05 |
|  | **DPC** | WT | 7.44 ± 0.07 | 0.69 ± 0.08 |
|  |  | E108Q | 7.78 ± 0.02 | 0.91 ± 0.04 |

**Table S2.** pKa values for WT PR and PR E108Q**,** as found from analysis of the maximum wavelength of absorption (shown in Figure S4B and C) and fits to a modified Henderson-Hasselbalch equation (Equation S1, indicated below in section S9)

| oligomeric state | detergent | mutant | A | B | pKa | n |
| --- | --- | --- | --- | --- | --- | --- |
| Oligomer | **DDM** | WT | 539.5 ± 0.8 | 22 ± 1 | 6.49 ± 0.07 | 0.70 ± 0.08 |
|  |  | E108Q | 557 ± 8 | 40 ± 11 | 6.4 ± 0.4 | 0.5 ± 0.2 |
|  | **DPC** | WT | 540 ± 2 | 24 ± 2 | 6.6 ± 0.1 | 0.6 ± 0.1 |
|  |  | E108Q | 547 ± 2 | 30 ± 2 | 6.5 ± 0.1 | 0.61 ± 0.07 |
| Monomer | **DDM** | WT | 542 ± 2 | 31 ± 4 | 7.4 ± 0.2 | 0.6 ± 0.2 |
|  |  | E108Q | 546 ± 2 | 33 ± 3 | 7.7 ± 0.1 | 1.1 ± 0.3 |
|  | **DPC** | WT | 534.0 ± 0.5 | 23.0 ± 0.9 | 7.83 ± 0.05 | 1.0 ± 0.1 |
|  |  | E108Q | 534.6 ± 0.9 | 29 ± 1 | 7.48 ± 0.06 | 1.1 ± 0.2 |

**(S5) Time-resolved optical absorption of PR E108Q in DPC and diC7PC**

**
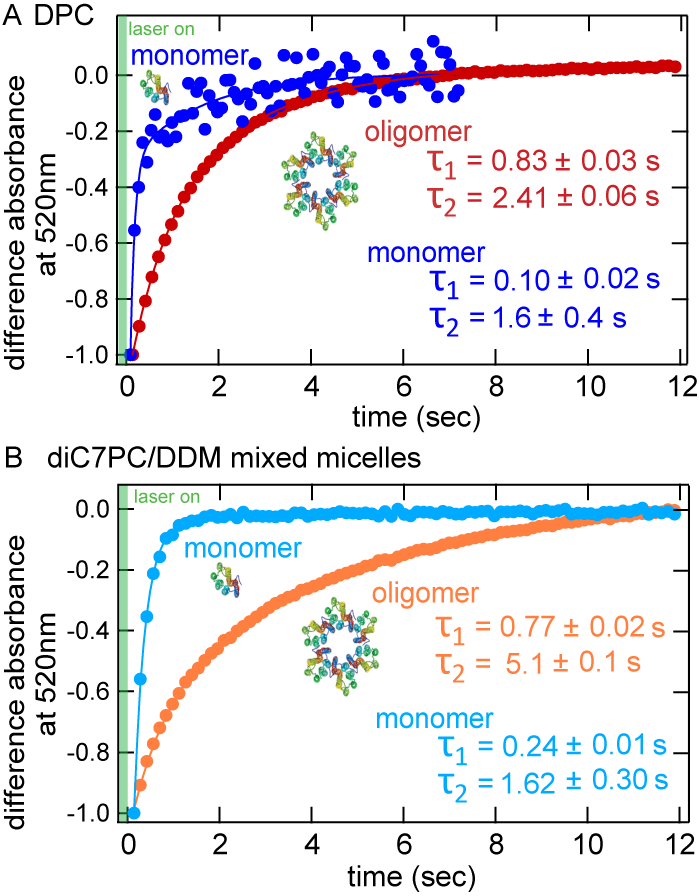
** In order to supplement the time-resolved optical absorption of PR E108Q in DDM shown in Figure 3 of the main text, which shows a clear dependence of M state decay with oligomeric state, the same measurements were repeated in the other two surfactant systems studied: DPC, and diC7PC. For both cases, the general trend of the oligomer having slowed photocycle kinetics relative to the monomer was upheld, as seen with DDM, but the absolute timescales found from the biexponential fit to the M state decay (τ_1_ and τ_2_) were found to vary with detergent type (Figure S5). We must note that monomeric PR in DPC was not stable at the pH chosen for the measurement, and therefore a short measurement time with minimal signal averaging was used (Figure S5A).

**Figure S5.** Time-resolved optical absorption measurement of E108Q PR at pH 9.8, indicating the decay of the M photointermediate for both the oligomeric and monomeric form of PR in (A) DPC and (B) diC7PC.

**(S6) Supplemental EPR, details of DSS crosslinking, and functional measurements of crosslinked PR**

The low-temperature EPR spectra of PR spin-labeled at the oligomeric interface (site 55 on the A-B loop) reveal that the distance between this site on adjacent PR molecules in the oligomer is extended for the DPC case relative to the DDM case, such that the dipolar broadening effect evident in DDM disappears (main text, Figure 6). The dipolar broadening for oligomeric PR in DDM is also observable with room-temperature EPR (Figure S6), but in DPC, the narrow linewidth and prominent contribution of the mobile component (labeled “*m*” in Figure S7) indicates that site 55 is now part of a rather flexible protein segment that is not in very close proximity (<20 Å) to the same 55 residue in the adjacent PR. Similarly, the low-temperature EPR measurement of the PR oligomer crosslinked in DSS (main text, Figure 6) provided evidence for a spatially expanded oligomeric structure, which is visible in the room-temperature spectra, where similar lineshapes are visible for crosslinked PR in either DDM or DPC (Figure S6). Spectra of monomeric PR in the two surfactants are also shown.


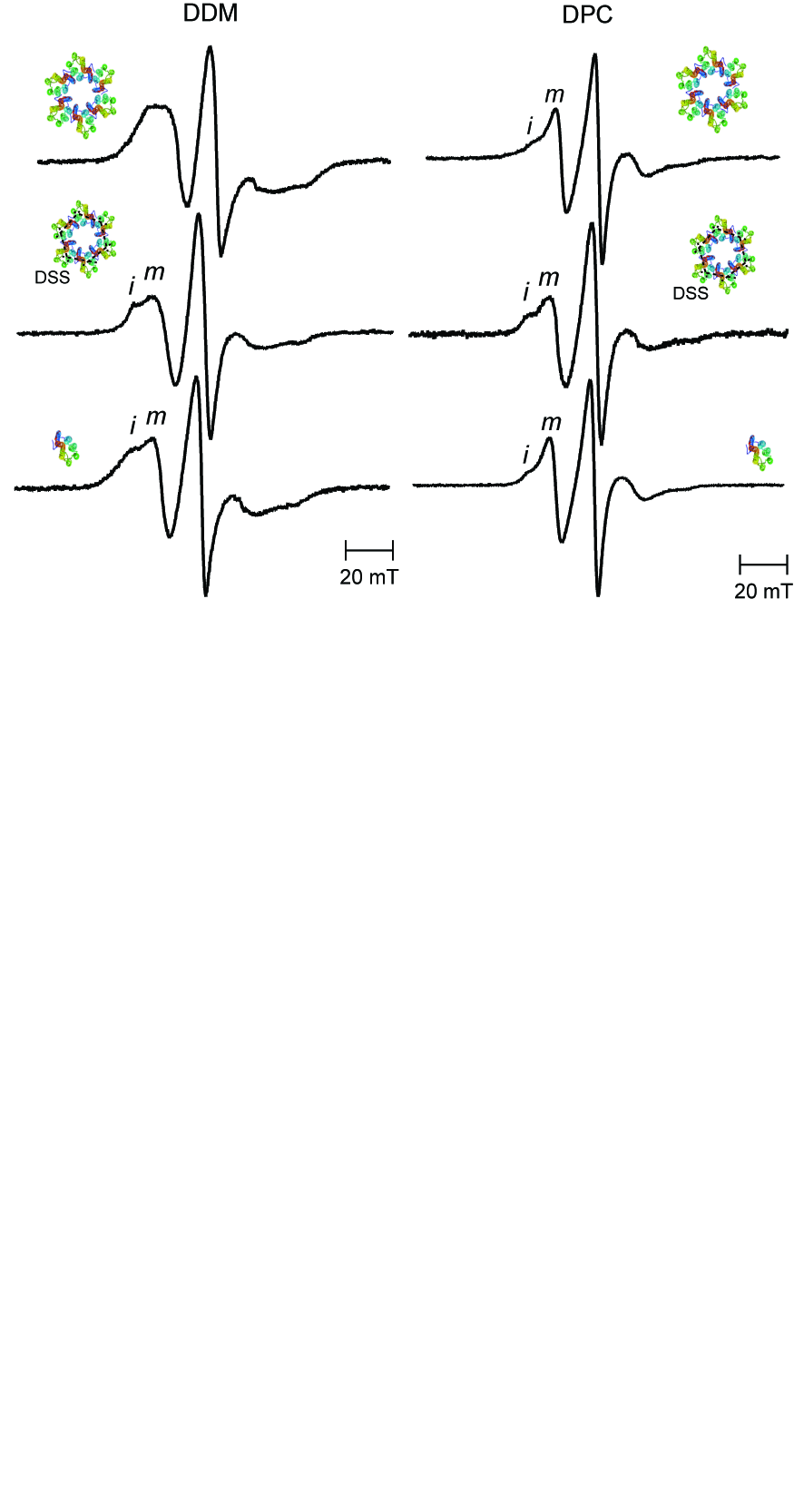
In order to understand the crosslinking phenomena utilized extensively by this study, we applied developed computational tools that predict amino acid residues that could be involved in possible crosslinking reactions ^13^. Using the blue-PR (B-PR) crystal structure as a template, analysis of the solvent accessible distance ^13^ across the hexamer, combined with the distance range of the DSS crosslinker as found from simulation (5.58 Å-11.42 Å^14^, corresponding to a maximum crosslinked CB-CB distance of 21.4 Å after taking into account the length of the lysine side chain) suggests that the most likely crosslink is between a lysine in the B helix (K57, homologous to B-PR residue K39) and the lysine in the C-terminal tail of an adjacent PR (K244, homologous to B-PR residue K226). There are no obvious predictions for intra-PR crosslinks, consistent with the apparent maintained folding of PR upon crosslinking, as evident from preserved functional properties (Figure S7). A second possible crosslinking site exists in the B helix at another residue, K59 (homologous to B-PR residue K41) with lysine K244 in the C-terminal tail of an adjacent PR if the K57-K244 crosslink is not formed. However, we note that the K59-K244 contact is further apart than the K57-K244 contact in the hexamer as calculated by solvent accessible distances—20.1 Å apart for K59-K244 compared to 14.9 Å for K57-K244 case. In either case, considering that protein dynamics, lateral movement of PRs within the membrane, and the flexibility of the crosslinker itself are all variables that could skew the end result, there is likely only one crosslink per PR between the B helix of one PR and the C-terminal tail of the adjacent PR, as depicted in the main text, Figure 4.

**Figure S6.** Room-temperature cw EPR spectra of a nitroxide spin label side-chain incorporated on A-B loop site 55 of PR in DDM and DPC in both oligomeric and monomeric forms, as well as the oligomer crosslinked with DSS.


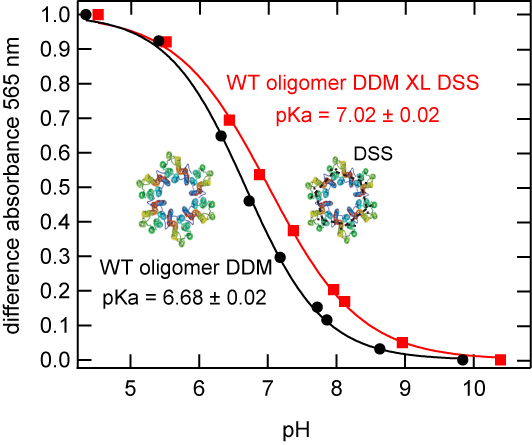
Functional measurements of PR crosslinked with DSS and then purified in its oligomeric state revealed small changes compared to the uncrosslinked, WT protein (Figure S7), especially compared to the large changes observed upon altering the oligomeric state (main text, Figures 3 and 4). An example of this is given in the pKa measurement of uncrosslinked and crosslinked PR shown in Figure S8, where the shift of ~0.3 pH points is far less than upon abolishing all oligomeric contacts by isolation of monomer (main text, Figure 3).

**Figure S7.** pH-dependence of the optical absorption spectra of WT PR, both when crosslinked (XL) with DSS, and without crosslinking, as well as the corresponding D97 pKa found from fits to the Henderson-Hasselbalch equation (Equation S2).

**(S7) Functional measurements of spin-labeled PR**


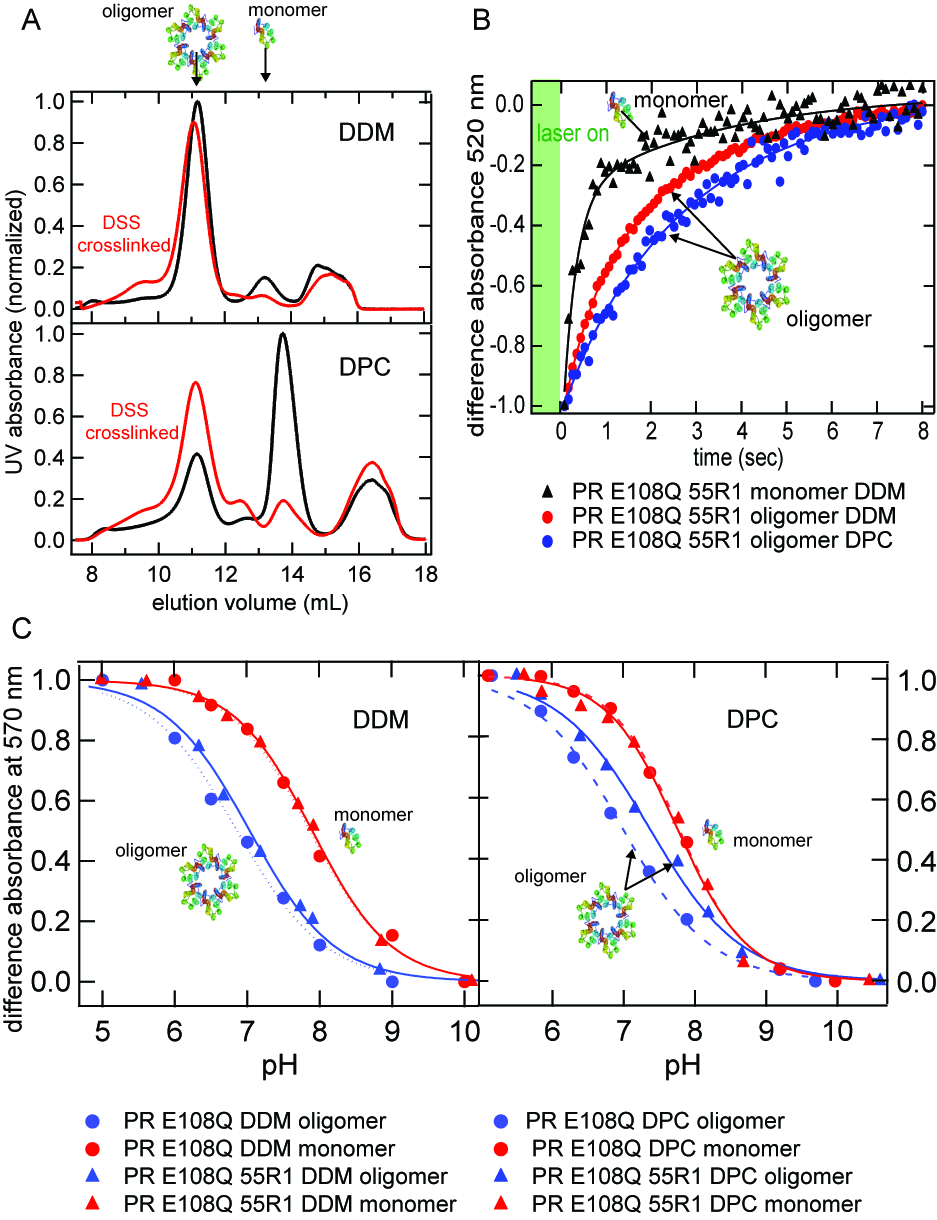
Since PR spin-labeled at the 55 site was used to characterize the PR oligomer in various surfactants and upon crosslinking (main text, Figure 6), we sought to check that the isolation of oligomeric states and the resultant functional properties were maintained for the spin-labeled mutant and further, upon crosslinking. We did not see a significant change in the oligomeric forms isolated by SEC (Figure S8A) upon spin-labeling, but as would be expected, there is a prominent change in the oligomeric distribution in DPC upon purifying the crosslinked PR, enriching the form identified to be hexameric in Stone, et. al.^2^ Furthermore, the presence of the oligomeric and monomeric forms of PR even after crosslinking with a large excess of crosslinking agent suggests that there may be a distribution of oligomeric states within the cell membrane itself (Figure S8A). The trends of oligomer-dependent photocycle kinetics (Figure S8B) and pKa values were also upheld upon spin-labeling, although for DPC there did appear to be a shift in pKa from wild-type (Figure S8C).

**Figure S8.** (A) SEC chromatograms of PR E108Q 55R1 in DDM and DPC, and with DSS crosslinking, and the corresponding functional measurements of (B) time-resolved optical absorption tracking of M state decay, and (C) pH-dependent optical absorption properties.

**(S8) Further details of Materials and Methods**

**Expression, purification, and separation of oligomeric forms of PR in surfactant complexes**

The expression and purification of cysteine-free green-absorbing PR, both with and without the E108Q mutation that enriches the M state of PR, was largely carried out similarly to as in previous studies.^2; 15^ Some variations include the cloning of the wild-type and E108Q PR genes with 6xHis tags into a kanamycin-resistant pET26b vector to better control the expression of PR in BL21(DE3)-pLysS cells (Agilent). After purification of PR and overnight incubation in a base buffer (50 mM potassium phosphate, 150 mM KCl, pH 8.2) containing 2 wt% of the chosen detergent (DDM, DPC, or diC7PC), SEC chromatography was conducted as described in Stone, et. al^2^ using a lower concentration of the surfactant of choice (.05 wt% DDM and 0.08 wt% DPC). The fractions corresponding to oligomeric and monomeric forms were then pooled and concentrated using either 50-kDa (for DDM) or 10-kDa MWCO centrifugal filters (Millipore).

**Crosslinking and SDS-PAGE**

Crosslinking of inter-PR lysines using DSS was performed similarly to as reported in the literature.^16^ A small volume of DSS in a freshly-made 150 mM dimethylsulfoxide (DMSO) solution was added in large molar excess (20-100 times) relative to the amount of PR in the base phosphate buffer, quantified by optical absorption,^17^ such that the final DSS concentration was 2 mM. After incubation with nutation for one hour at room temperature, the crosslinking reaction was quenched by addition of Tris to a final concentration of 50 mM. The same procedure was used to crosslink purified PR and PR in *E. coli* membranes extracted by ultracentrifugation (220,000 g) after cell lysis.

SDS-PAGE was performed using a Bio-Rad electrophoresis apparatus with Criterion TGX Stain-Free gels having an 8-16% acrylamide gradient (Bio-Rad). Samples containing ~3μg PR were incubated at 37 ̊C for 30 minutes to 1 hour with Laemmli buffer (final SDS concentration of 1%), and then centrifuged to remove the unsolubilized portions. The gel was run at 120 V for one hour, and imaged with a ChemiDoc MP imaging system (Bio-Rad) using a PR-specific protocol (excitation with green epi light and 695/705 filters to detect emission) based on the fluorescence properties of PR ^17^. Protein markers (Precision Plus, Bio-Rad) and total proteins were further imaged using the Stain-Free protocol of ChemiDoc MP. Molecular weight estimates were calculated using the ImageLab software (Bio-Rad).

**Optical absorption spectroscopy**

The pH-dependent optical absorption spectra of wild-type PR and the variants studied here were measured with a Shimadzu UV-1800 spectrophotometer. A concentrated (~100-800 μM) solution of PR was diluted by a factor of 20-100 in the base phosphate buffer supplemented with the appropriate amount of surfactant (.05 wt% DDM or 0.08 wt% DPC) at the target pH. The final pH of the diluted PR solution was then measured with a micropH electrode (Wilmad) for accuracy. In order to calculate the pKa of D97, two methods were used, as described in section S3, one using the absolute measure of color, maximum wavelength of absorption, and another using difference spectra relative to the most alkaline condition. For fitting the maximum wavelength of absorption (*λ_max_*) as a function of pH, a modified Henderson-Hasselbalch equation was used as shown below, where the measured *λ_max_* was plotted against pH, and the fit parameters *A* and *B* calculated with fitting software (Igor Pro), as well as the *pKa* and cooperativity parameter, *n*.

$pH=A+ \frac{B}{1+{10}^{n(\lambda\max-pKa)}}$ Equation S1

Similarly, a second method involved subtracting the most basic of the spectra taken (resulting in difference spectra such as those shown in Figure S3B), and plotting the absorbance (*diffAbs*) at the maximum of these difference spectra (often at 565-570 nm) against the pH. The *pKa* and cooperativity parameter (*n*) could then be fit using the equation:

$pH= \frac{1}{1+{10}^{n(diffAbs-pKa)}}$ Equation S2

A home-built time-resolved optical absorption spectrometer, as described in a prior study ^15^, was used to observe the photocycle kinetics for the slowed-photocycle E108Q mutant. Snapshots of the difference spectrum upon activation were taken ~80 ms apart in the visible range (400 nm to 700 nm). These measurements were taken at an appropriately alkaline pH (9.8) as chosen from the pKa curves (Figure S3), so as to be working at a point where the relative populations of the deprotonated PR form would be similar for both the oligomer and the monomer.

**Electron Paramagnetic Resonance (EPR) spectroscopy**

The slowed-photocycle S55C mutant was spin-labeled as described in a prior study ^2^, and studied with cw X-band (0.35 T) EPR measurements. Room-temperature spectra were taken of 4 μL samples (100-800 μM PR) in quartz capillaries (outer diameter 0.84 mm, inner diameter 0.6 mm, Vitrocom) using a Bruker EMXplus equipped with the ER 4119HS-LC High Sensitivity Probehead (Bruker). Spectral parameters were set to 20 mW microwave power, 1 G modulation amplitude, and 150 G total sweep width. Low-temperature spectra were measured at 140 K with the greater modulation amplitude of 2 G, as well as a 200 G total sweep width.

**(S9) Supporting References**

1. Rath, A., Glibowicka, M., Nadeau, V. G., Chen, G. & Deber, C. M. (2009). Detergent binding explains anomalous SDS-PAGE migration of membrane proteins. *Proc Natl Acad Sci U S A* **106**, 1760-5.

2. Stone, Katherine M., Voska, J., Kinnebrew, M., Pavlova, A., Junk, Matthias J. N. & Han, S. (2013). Structural Insight into Proteorhodopsin Oligomers. *Biophysical Journal* **104**, 472-481.

3. Lipfert, J., Columbus, L., Chu, V. B., Lesley, S. A. & Doniach, S. (2007). Size and shape of detergent micelles determined by small-angle X-ray scattering. *J Phys Chem B* **111**, 12427-38.

4. Sanders, C. R. & Sonnichsen, F. (2006). Solution NMR of membrane proteins: practice and challenges. *Magn Reson Chem* **44 Spec No**, S24-40.

5. le Maire, M., Champeil, P. & Moller, J. V. (2000). Interaction of membrane proteins and lipids with solubilizing detergents. *Biochim Biophys Acta* **1508**, 86-111.

6. Warschawski, D. E., Arnold, A. A., Beaugrand, M., Gravel, A., Chartrand, E. & Marcotte, I. (2011). Choosing membrane mimetics for NMR structural studies of transmembrane proteins. *Biochim Biophys Acta* **1808**, 1957-74.

7. Prive, G. G. (2007). Detergents for the stabilization and crystallization of membrane proteins. *Methods* **41**, 388-97.

8. Oliver, R. C., Lipfert, J., Fox, D. A., Lo, R. H., Doniach, S. & Columbus, L. (2013). Dependence of micelle size and shape on detergent alkyl chain length and head group. *PLoS ONE* **8**, e62488.

9. Hauser, H. (2000). Short-chain phospholipids as detergents. *Biochimica Et Biophysica Acta-Biomembranes* **1508**, 164-181.

10. Reckel, S., Gottstein, D., Stehle, J., Löhr, F., Verhoefen, M.-K., Takeda, M., Silvers, R., Kainosho, M., Glaubitz, C., Wachtveitl, J., Bernhard, F., Schwalbe, H., Güntert, P. & Dötsch, V. (2011). Solution NMR Structure of Proteorhodopsin. *Angewandte Chemie International Edition*, 11942–11946.

11. Yamada, K., Kawanabe, A. & Kandori, H. (2010). Importance of alanine at position 178 in proteorhodopsin for absorption of prevalent ambient light in the marine environment. *Biochemistry* **49**, 2416-23.

12. Sharaabi, Y., Brumfeld, V. & Sheves, M. (2010). Binding of Anions to Proteorhodopsin Affects the Asp97 pKa. *Biochemistry* **49**, 4457–4465.

13. Kahraman, A., Malmstrom, L. & Aebersold, R. (2011). Xwalk: computing and visualizing distances in cross-linking experiments. *Bioinformatics* **27**, 2163-2164.

14. Green, N. S., Reisler, E. & Houk, K. N. (2001). Quantitative evaluation of the lengths of homobifunctional protein cross-linking reagents used as molecular rulers. *Protein Science* **10**, 1293-1304.

15. Hussain, S., Franck, J. M. & Han, S. (2013). Transmembrane Protein Activation Refined by Site-Specific Hydration Dynamics. *Angewandte Chemie International Edition* **52**, 1953-1958.

16. Mattson, G., Conklin, E., Desai, S., Nielander, G., Savage, M. D. & Morgensen, S. (1993). A Practical Approach to Cross-Linking. *Molecular Biology Reports* **17**, 167-183.

17. Lenz, M. O., Huber, R., Schmidt, B., Gilch, P., Kalmbach, R., Engelhard, M. & Wachtveitl, J. (2006). First Steps of Retinal Photoisomerization in Proteorhodopsin. *Biophysical Journal* **91**, 255 - 262.
